# The mechanism of action of the adrenomedullin-binding antibody adrecizumab

**DOI:** 10.1186/s13054-018-2074-1

**Published:** 2018-06-13

**Authors:** Christopher Geven, Peter Pickkers

**Affiliations:** 1 Department of Intensive Care Medicine, Radboud University Medical Center, PO Box 9101, Internal Mail 710, Nijmegen, 6500 HB The Netherlands; 2 Radboud Center for Infectious Diseases, Radboud University Medical Center, Nijmegen, The Netherlands

With interest we read the review by Levy et al. [1] that provides a thorough overview of current and future therapies aiming to treat vasoplegia, an ubiquitous phenomenon in shock. In their work, the authors dedicated a section to the novel adrenomedullin (ADM)-binding antibody adrecizumab, which is mentioned as an *ADM blocking* compound in the section title. Recently published studies demonstrate that blocking of ADM does not accurately describe adrecizumab’s mechanism of action, though.

In contrast to what is often intuitively assumed, not all antibodies completely inhibit the activity of their targets. The extent of signaling inhibition can vary greatly, depending on the epitope to which the antibodies bind and other factors, such as antibody concentrations. In contrast to C-terminus binding anti-ADM antibodies which completely inhibit ADM signaling, antibodies against the N-terminus of ADM, including the humanized monoclonal antibody adrecizumab, only *marginally* inhibit ADM activity, despite their high affinity and even when applied in vast molar excess over ADM [2].

Interestingly, animal and human data reveal a strong, dose-dependent *increase* of plasma ADM concentrations upon adrecizumab infusion [3, 4], which cannot be explained by increased production of ADM. A mechanistic explanation for this occurrence was recently proposed [5]. Excess antibody that remains in the circulation is thought to drain ADM from the interstitium into the circulation, since ADM is small enough to cross the endothelial barrier, whereas the antibody is not. While adrecizumab only partially inhibits ADM signaling, the strong concentration increase of ADM (complexed with adrecizumab) in the circulation is thought to result in an overall “net” increase of ADM activity on endothelial cells, augmenting endothelial barrier stabilizing effects, while decreased concentrations of ADM in the interstitium reduce vasodilatory effects on vascular smooth muscle cells. The combination of attenuation of both endothelial leakage and vasodilation likely represents the therapeutic potential of adrecizumab. This hypothesis fits well with previous studies that showed beneficial effects of ADM agonists in animal models of shock, while complete inhibition of ADM did not improve outcome.

As is mentioned by the authors, a proof-of-concept and dose-finding phase II study with adrecizumab is currently ongoing in patients with septic shock and elevated levels of ADM (ClinicalTrials.gov identifier NCT03085758). This trial incorporates a number of innovative features such as a novel composite efficacy endpoint and biomarker-guided patient selection (enrolment based on bio-ADM level), making it one of the first personalized treatment trials in sepsis.

## Abbreviation

ADM: Adrenomedullin

## References

[1] Levy B, Fritz C, Tahon E, Jacquot A, Auchet T, Kimmoun A (2018). Vasoplegia treatments: the past, the present, and the future. Crit Care.

[2] Struck J, Hein F, Karasch S, Bergmann A (2013). Epitope specificity of anti-Adrenomedullin antibodies determines efficacy of mortality reduction in a cecal ligation and puncture mouse model. Intensive Care Med Exp..

[3] Geven C, Peters E, Schroedter M, Struck J, Bergmann A, McCook O, et al. Effects of the humanized anti-adrenomedullin antibody adrecizumab (HAM8101) on vascular barrier function and survival in rodent models of systemic inflammation and sepsis. Shock. 2018; 10.1097/SHK.0000000000001102. 10.1097/SHK.0000000000001102 29324627

[4] Geven C, van Lier D, Blet A, Peelen R, ten Elzen B, Mebazaa A, et al. Safety, tolerability and pharmacokinetics/-dynamics of the adrenomedullin antibody Adrecizumab in a first-in-human study and during experimental human endotoxemia in healthy subjects. Br J Clin Pharmacol. 2018; 10.1111/bcp.13655. 10.1111/bcp.13655 PMC6089825 29856470

[5] Geven C, Bergmann A, Kox M, Pickkers P. Vascular effects of Adrenomedullin and the anti-adrenomedullin antibody adrecizumab in sepsis. Shock. 2018; 10.1097/SHK.0000000000001103. 10.1097/SHK.0000000000001103 29324626

